# Deterministic and stochastic modelling of impacts from genomic selection and phenomics on genetic gain for perennial ryegrass dry matter yield

**DOI:** 10.1038/s41598-021-92537-w

**Published:** 2021-06-24

**Authors:** M. Z. Z. Jahufer, Sai Krishna Arojju, Marty J. Faville, Kioumars Ghamkhar, Dongwen Luo, Vivi Arief, Wen-Hsi Yang, Mingzhu Sun, Ian H. DeLacy, Andrew G. Griffiths, Colin Eady, Will Clayton, Alan V. Stewart, Richard M. George, Valerio Hoyos-Villegas, Kaye E. Basford, Brent Barrett

**Affiliations:** 1 Grasslands Research Centre, AgResearch Ltd, Palmerston North, 4442 New Zealand; 2 School of Agriculture and Food Sciences, Faculty of Science, The University of Queensland, Brisbane, QLD 4072 Australia; 3 Barenbrug NZ Ltd, Christchurch, 7571 New Zealand; 4 Kimihia Research Centre, PGG Wrightson Seeds Ltd, Christchurch, 7676 New Zealand; 5 Department of Plant Science, McGill University, Montreal, QC H3A 0G4 Canada; 6 The Centre for Complex Analytics and Visualisation, AutoStat Institute, Melbourne, VIC 3000 Australia

**Keywords:** Genetics, Plant sciences

## Abstract

Increasing the efficiency of current forage breeding programs through adoption of new technologies, such as genomic selection (GS) and phenomics (*Ph*), is challenging without proof of concept demonstrating cost effective genetic gain (∆G). This paper uses decision support software DeltaGen (tactical tool) and QU-GENE (strategic tool), to model and assess relative efficiency of five breeding methods. The effect on ∆G and cost ($) of integrating GS and *Ph* into an among half-sib (HS) family phenotypic selection breeding strategy was investigated. Deterministic and stochastic modelling were conducted using mock data sets of 200 and 1000 perennial ryegrass HS families using year-by-season-by-location dry matter (DM) yield data and in silico generated data, respectively. Results demonstrated short (deterministic)- and long-term (stochastic) impacts of breeding strategy and integration of key technologies, GS and *Ph*, on ∆G. These technologies offer substantial improvements in the rate of ∆G, and in some cases improved cost-efficiency. Applying 1% within HS family GS, predicted a 6.35 and 8.10% ∆G per cycle for DM yield from the 200 HS and 1000 HS, respectively. The application of GS in both among and within HS selection provided a significant boost to total annual ∆G, even at low GS accuracy r_A_ of 0.12. Despite some reduction in ∆G, using *Ph* to assess seasonal DM yield clearly demonstrated its impact by reducing cost per percentage ∆G relative to standard DM cuts. Open-source software tools, DeltaGen and QuLinePlus/QU-GENE, offer ways to model the impact of breeding methodology and technology integration under a range of breeding scenarios.

## Introduction

Designing the best structure for a breeding program, within the resources available, are important decisions to be made at the onset of cultivar development^1,2^. In forage breeding, there are a range of well proven and commonly used methods based on half-sib and full-sib family recurrent selection^3,4^, which have often been adapted to suit specific breeding objectives. Today, genomic selection and marker-aided breeding approaches^5–7^ together with high throughput, non-destructive phenotyping platforms^8–11^ provide new opportunities for plant breeders to improve the efficiency and accuracy of conventional plant breeding strategies.

The cost and time required for phenotypic assessment limits the efficiency of screening traits in crops and forages. This is particularly the case for evaluating yield or stress related traits such as DM yield, increased tolerance to abiotic stress, or water use efficiency^12^. Breeding focused on improving growth is often constrained by destructive methods of biomass measurement across multiple genotypes evaluated in field trials^13^. The application of powerful sensors and digital tools can help in accurate trait quantification^14^. In the past five years adoption of these phenomics based technologies and tools in crops and forages has increased markedly^8,11,14–17^.

Questions associated with optimisation of breeding strategies are complex, compounded by cost and uncertainty of changing a system, even if the existing system may be delivering less genetic gain, be less nimble or address fewer traits than desired. Decision support software enables simulation of breeding strategy efficacy in response to factors including selection among and within genetic families, different combinations of year, season, site and replicate with associated costs per selection cycle. Such software help breeders design and implement more efficient and effective breeding programs based on predicted genetic gain. To date this has been focused on simulation for inbred species^18–20^, leaving a gap in obligate outcrossing species such as forage grasses. Recent efforts to address this gap have led to the development of two decision support software packages for plant breeding: the tactical tool DeltaGen^21^ based on deterministic modelling; and the strategic tool QuLinePlus^22^ a component of QU-GENE^18^ based on stochastic modelling. QuLinePlus is a breeding module, specifically designed to simulate full and half sibling breeding programs for outcrossing species and QU-GENE is considered as an engine which generates the simulation inputs. Concurrently, new phenomic tools for electronic automation, acceleration and standardisation of data collection for key traits such as DM yield have been developed^8^, and are being deployed in field breeding programs^23^. Genomic prediction models have recently been developed and assessed for complex traits, including DM yield^24^ and nutritive value^25^ in perennial ryegrass (*Lolium perenne*). Understanding the implications of these new technologies on genetic gain over time is pivotal to ensuring their use is optimized.

The availability of *Ph* and GS tools for forage plant breeding, and of decision support simulation tailored for outbred species, gives rise to the opportunity to examine the relative costs and impact of options for integrating these tools into breeding systems. This paper aims to investigate, using modelling and simulation, the relative efficiency of five half sib (HS) breeding methods based on predicted genetic gain and associated costs to improve DM yield of perennial ryegrass. Simulation was conducted applying tactical deterministic and strategic stochastic decision support modelling software (i.e. DeltaGen and QU-GENE) based on a “mock data” set of 1000 HS families, created by combining DM yield data from two sets of field trials. A key objective of this paper is also to demonstrate the potential application of the combination of DeltaGen/QU-GENE, to expand the scope of tools available to breeders in decision support for breeding program design.

The five HS family breeding methods simulated were: (a) standard phenotypic among half-sib family selection (A_*p*_), (b) among half-sib family selection based on phenomics (A_*Ph*_), (c) standard phenotypic among half-sib family selection and within family genomic selection (A_*p*_W_*gs*_), (d) among half-sib family selection based on phenomics and within family genomic selection (A_*Ph*_W_*gs*_), (e) both among and within half-sib family selection based on genomic selection (A_*gs*_W_*gs*_).

## Material and methods

### Deterministic modelling for estimating genetic gain using HS family breeding strategies

The genetic gain simulation analyses were conducted using the open source software DeltaGen^21^ (available via https://deltagen.agresearch.co.nz/app/deltagen) for single selection cycles. The constructed mock data matrices of 200 HS and 1000 HS families used to generate starting points for the simulation of breeding methods, were compiled using perennial ryegrass DM yield and growth score measurements generated from two sources of multi-year-season-location trials. The term *starting points* referred to in this paper, are a common set of HS family means, estimates of additive genetic/interaction variance components and narrow sense heritabilities, applied in the different breeding equations used for deterministic modelling and simulation of the five breeding methods using DeltaGen.

#### Construction of the 1000 and 200 HS family “mock data” matrices of perennial ryegrass

Prediction of differences in genetic gain ∆G among breeding strategies is based on the application of quantitative genetic models^3,4^. These genetic analyses require estimates of population parameters; phenotypic variance (${\sigma }_{P}^{2}$), additive genetic variance (${\sigma }_{A}^{2}$), different components of genotype-by-environment (GE) interaction such as HS family interactions with; year, season and location, depending on breeding objectives, and narrow sense heritability (${h}_{n}^{2}$). In our investigation using deterministic simulation, a key criterion for modelling the impact of GS and *Ph* technologies on the relative efficiency of HS family breeding methods, was to use mock data sets constructed from actual HS family multi location- year-season field trials rather than using in silico generated data. These mock data matrices enabled “realistic” estimates of genetic parameters to be generated. It is important to note that these estimates were only used as starting points to conduct simulation of the breeding methods using DeltaGen. Two mock DM yield data matrices, of 1000 HS and 200 HS families, were created by combining data generated from multi-location, year and season, HS family evaluation studies reported by Faville et al.^24^ and Arojju et al.^26^.

The 1000 HS family DM yield mock data set was based on field trials conducted across 2 locations over 2 years and 3 seasons per year. The mock data set was constructed so that each location had a 20 row-by-50 column design with 3 replicates. The herbage DM yield data matrix consisting of 200 HS families was constructed from a random sample of 200 families taken from the 1000 HS family matrix. The year ($n$=2), season ($n$=3) and location ($n$=2) data associated with each of the 200 randomly sampled HS families, were combined into a 3 replicate, row and column (10 rows- by-20 columns) by location, season and year design matrix. A detailed description of construction of the two mock data sets is provided in supplementary material 1 in File S1, and the distribution of the 200 and 1000 HS family DM yield data are presented in Figure S1.

It is important to note that a key assumption used for quantitative genetic analysis and modelling of the mock data, was that both the 200 HS and 1000 HS families were generated from parents randomly sampled from the same random mating population, cross pollinated under isolation. The coefficient of inbreeding ($F$) was assumed to be zero $(F=0)$ and therefore the among HS family (${\sigma }_{f}^{2}$) variation provided an estimate of ¼ ${\sigma }_{A}^{2}$ (additive genetic variation)^27^.

#### Data analysis

The two HS family mock data matrices (provided as supplementary material 2) were analysed using Eqs. 1 and 2 below, to derive estimates of additive genetic variation, associated genotype-by-environment (GE) interaction and narrow sense heritability on a family mean basis. A linear mixed model (Eq. 1) using the Residual Maximum Likelihood (REML)^28–30^ procedure in DeltaGen^21^ was used to estimate genetic parameters to be used for modelling based on breeding equations.

The linear mixed model used for analysis across locations, seasons and years:

1 $$Y_{{ijklmno}} = M + f_{i} + l_{j} + \left( {fl} \right)_{{ij}} + s_{k} + \left( {fs} \right)_{{ik}} + y_{l} + \left( {fy} \right)_{{il}} + b_{{jklm}} + ~r_{{jklmn}} + c_{{jklmno}} + \varepsilon _{{ijklmno}}$$

${Y}_{ijklmno}$ is the value of an attribute measured from HS family $i$ in column $o$ and row $n$ of replicate $m$ at location $j$ in season $k$ of year $l$, and $i=$ 1,…, ${n}_{f}$; $j=$ 1,…,*n*_*l*_, $k=1$,…,${n}_{s}$; $l=1$,…, ${n}_{y}$; $m=$ 1,…, ${n}_{b}$; $n=1$,…, ${n}_{r}$; $o=1$,…,${n}_{c}$; where *f*, $l$, $s$, $y$, $b$, $r$ and $c$, are families, locations, seasons, years, replicates, rows and columns, respectively; $M$ is the overall mean; ${f}_{i}$ is the random effect of family $i$, $N(0, {\sigma }_{f}^{2});$ ${l}_{j}$ is the fixed effect of location $j$; ${\left(fl\right)}_{ij}$ is the effect of the interaction between family $i$ and location $j$, $N(0,{\sigma }_{fl}^{2})$; ${s}_{k}$ is the fixed effect of season $k$; ${\left(fs\right)}_{ik}$ is the effect of the interaction between family $i$ and season $k$, $N(0,{\sigma }_{fs}^{2})$; ${y}_{l}$ is the fixed effect of year $l$;${\left(fy\right)}_{il}$ is the effect of the interaction between family $i$ and year $l$, $N(0,{\sigma }_{fy}^{2})$; ${b}_{jklm}$ is the random effect of replicate $m$ within location $j$, within season $k$, within year $l$, $N(0,{\sigma }_{b}^{2})$; ${r}_{jklmn}$ is the random effect of row $n$ in replicate $m$ within location $j$, within season $k$, within year $l$, $N(0,{\sigma }_{r}^{2})$; ${c}_{jklmno}$ is the random effect of column $o$ in row $n$ in replicate $m$ within location $j$, within season $k$, within year $l$, $N(0,{\sigma }_{c}^{2})$; ${\varepsilon }_{ijklmno}$ is the residual effect for family $i$ in row $n$ and column $o$ in replicate $m$ in location $j$ in season $k$, during year $l$*,* $N(0,{\sigma }_{\varepsilon }^{2})$.

The estimates of variance components generated from Eq. 1, were used to generate an estimate of narrow sense heritability, ${h}_{n}^{2}$, for herbage DM yield on a family mean basis using Eq. 2.

2 $$h_{{n~}}^{2} = \frac{{\sigma _{f}^{2} }}{{\sigma _{f}^{2} + \frac{{\sigma _{{fl}}^{2} }}{{n_{l} }} + \frac{{\sigma _{{fs}}^{2} }}{{n_{s} }} + \frac{{\sigma _{{fy}}^{2} }}{{n_{y} }} + \frac{{\sigma _{\varepsilon }^{2} }}{{n_{s} n_{y} n_{r} }}}}$$

where $n$, number of and *f*, *l*, *s*, *y and r*, are families, locations, seasons, years and replicates, respectively.

DeltaGen generated estimates of narrow sense heritability (${h}_{n}^{2}$) on a HS family mean basis across locations, seasons and years^31^.

#### Breeding methods and associated prediction equations

The five breeding methods assessed in this study were:

- Standard phenotypic among HS family selection (A_*p*_): Elite HS families are selected based on phenotypic performance within or across environments. Equal numbers of remnant seed from each selected HS family are randomly sampled. The selected individuals become the parents of the next generation. The prediction equation for genetic gain^4^,
  3 $$\Delta G={k}_{f}c\frac{\frac{1}{4}{\sigma }_{A}^{2}}{{\sigma }_{PF}}$$
  where, ${k}_{f}$, is among family selection intensity; $c$, parental control factor; ${\sigma }_{A}^{2}$, additive genetic variance;${\sigma }_{PF}$, the phenotypic standard deviation among families. For HS family selection c = 0.5 as selection is on female gametes only^27^.
- Among HS family selection using LiDAR (light detection and ranging) based phenomics (A_*Ph*_): as per (a), elite HS families are selected but based on phenotypic data collected using a LiDAR based phenomic tool^8^. The precision of genetic gain estimated using breeding Eq. (3) will depend on the accuracy of the phenomics method applied.
- Standard phenotypic among HS family selection and within HS family genomic selection (A_*p*_W_*gs*_): This breeding method consists of the steps: (i) select elite HS families, using a predetermined selection pressure, based on standard phenotypic measurements as per (a); (ii) equal numbers of remnant seed from each selected HS family are randomly sampled, seedlings established and individually genotyped, individual genomic-estimated breeding values (GEBV’s) determined using genomic prediction, and the best seedlings within each family based on their GEBV’s are selected on a predetermined selection pressure; (iii) the selected individuals become the parents of the next generation. The genomic prediction model used in step (ii) is itself derived using standard phenotypic measurements from the HS field trial and DNA marker data from the maternal parents of the HS families^24,32,33^. The equation for predicting genetic gain,
  4 $${\mathrm{A}}_{p}{\mathrm{W}}_{gsY}={k}_{f}{c}_{f}\frac{\frac{1}{4}{\sigma }_{AY}^{2}}{{\sigma }_{PF}}+{k}_{w}{c}_{w}{h}_{X}{r}_{A-XY}\frac{\surd 3}{2}{\sigma }_{AY}$$
  where, ${\mathrm{A}}_{p}{\mathrm{W}}_{gsY}$ is the predicted ∆G for trait *Y* using a combination of standard phenotypic among HS family selection and within HS family genomic selection; ${\sigma }_{AY}^{2}$, additive genetic variance for primary trait *Y*; ${\sigma }_{AY}$, standard deviation of additive genetic variance for primary trait $Y$; $\sqrt{\frac{3}{4}}=\frac{\sqrt{3}}{2}$; ${\sigma }_{PF}$, among HS family phenotypic standard deviation; ${k}_{f}$ and ${k}_{w}$, among and within family selection intensity, respectively; ${c}_{f}$ (= 0.5) and ${c}_{W}$ (= 0.5) among and within family parental controls, respectively; ${h}_{X}$ _,_ square root of heritability for secondary trait $X$; ${r}_{A-XY}$, genomic prediction accuracy – Pearson’s correlation between Phenotypic Estimated Breeding Values – BLUP’s $Y$ and GEBV’s $X$. In GS, the assumption is ${h}_{X}$ =1^34,35^. Please note that the subscript $gsY$ in Eqs. 4 and 5 is for genomic selection ($gs$) for trait $Y$. This is based on correlated response where, trait $Y$ (the true breeding value of an individual) is indirectly selected based on selection for trait $X$ (the GEBV of that individual).
- Among HS family selection using LiDAR based phenomics (*Ph*) and within family genomic selection (A_*Ph*_W_*gs*_): as per (c) except that elite HS families are selected based on data collected using a LiDAR based *Ph* tool.
- Both among and within HS family selection based on genomic selection (A_*gs*_W_*gs*_): This breeding method is implemented in cycle 2 using the HS families generated using the A_*p*_W_*gs*_ method (c). In this scenario, following application of A_*p*_W_*gs*_, a second cycle of selection is completed using only genomic prediction. To achieve among family selection based on GEBV’s, a predetermined number of seeds is randomly sampled from each HS family and the seedlings genotyped. The marker information is used in the already-established genomic prediction model to generate GEBV estimates and a mean GEBV for each family is determined. Based on the required selection pressure, HS families are selected on their GEBV means. Within-family selection of the chosen HS families, using a predefined selection pressure, is applied to family members based on their individual GEBV estimates, as per (c). The selected individuals form the parents of the next generation. The equation used for predicting genetic gain^21^,
  5 $${\mathrm{A}}_{gsY}{\mathrm{W}}_{gsY}={k}_{f}{c}_{f}{h}_{X}{r}_{A-XY}\frac{1}{2}{\sigma }_{AY}+{k}_{w}{c}_{w}{h}_{X}{r}_{A-XY}\frac{\surd 3}{2}{\sigma }_{AY}$$
  where the terms in the above equation have been defined in equation 4.

#### Genomic selection

For the purposes of this exercise, the cost of conducting GS was defined as the cost per GEBV. The costing was based on a non-commercial research laboratory, the Forage Genetics GBS facility at AgResearch, and includes consumables and time. All steps from extracting DNA through genotyping-by-sequencing (GBS) to generate a GEBV are addressed in supplementary material 1, Table S1: DNA isolation, GBS library development, GBS library sequencing, bioinformatic processing and genotype calling from raw GBS data, and genomic prediction (GEBV determination). Seedling grow-out and tissue sampling were not included as it is expected tissue samples for DNA isolation would be provided. The GBS process, including data filtering steps, is largely as described by Faville, et al.^24^ except that it is conducted at a 384-plex scale (376 samples plus four blanks and four positive controls per library) instead of 96-plex (94 samples plus one blank and one positive control).

#### Phenomics, sampling and field trial operational costs

The *Ph* referred to in this paper is a LiDAR based mobile platform for non‑invasive vegetative biomass and growth rate estimation in perennial ryegrass. The accuracy of 0.90 was determined from LiDAR based volumetric estimates compared against fresh weight and dry weight data across different ages of plants, seasons, stages of regrowth, sites, and row plot (two 2 m rows 15 cm apart) configurations^23^. The costs (New Zealand $) per sample; based on perennial ryegrass herbage DM derived via harvest and via *Ph* were $7.50 and $0.87, respectively. These sampling costs were obtained from multi-year, season and location field trials, based on row plots, which generated the original data used to build the 1000 HS family data matrix. The cost information is provided in supplementary material 1, Table S2.

### Stochastic modelling of multiple selection cycles using HS family breeding strategies

Stochastic modelling of the three breeding strategies A_*p*_, A_*p*_W_*gs*_ and A_*gs*_W_*gs*_ were conducted using QuLinePlus^22^, available via https://sites.google.com/view/qu-gene, with modifications to the software to implement GS. The objective of this modelling was to generate trends of response to selection over multiple breeding cycles, based on populations of similar size used in deterministic modelling.

The breeding strategies; A_*p*_, A_*p*_W_*gs*_ and A_*gs*_W_*gs*_ were simulated across ten selection cycles using QuLinePlus software. The three strategies were compared for percentage of genetic gain per year (%ΔG), cumulative genetic gain (ΣΔG), allele fixation rate, genetic variance and prediction accuracy across multiple selection cycles. Each generated value is an average 250 iterations.

#### Generation of the initial training population for simulation in QuLinePlus

Two sets of training populations consisting of 196 and 980 HS families, hereafter referred to as Sim200 and Sim1000, were simulated. Beginning with experimentally derived information on phenomic and associated genomic data from a commercial breeding population consisting of 98 plants, QuLinePlus software was used to generate 98 HS families. These HS families were used to simulate their performance for DM yield for three years across three locations. From each of the evaluated families two random individuals were drawn to generate a training population of 196 plants (Sim200) and ten random individuals were drawn to generate a second training population of 980 plants (Sim1000). It is important to note that the 200 and 1000 HS families used in deterministic modelling were related, the former a random sample from the latter.

#### Defining the genetic model and trait architecture

A recombination map with seven linkage groups was constructed based on a genetic map^36^ using the genomic markers from the initial population (98 individuals). In total, 1807 segregating markers were assigned to the seven linkage groups, out of which 474 were QTLs with an additive gene model. The allelic effect of each QTL was estimated based on genome-wide association analysis for DM yield implemented using GAPIT^37^.

Narrow sense heritabilities on a family mean basis, estimated from mixed model analysis in DeltaGen using data for mean DM yield of the simulated 200 HS and 1000 HS families, were converted to single plant-based heritabilities (considering 30 plants per plot).

#### Genomic prediction

A genomic prediction model was generated using marker and phenotypic data from the training populations, Sim200 and Sim1000. The GEBVs for each individual plant were estimated using a standard BLUP procedure using the R package rrBLUP^38^. The heritability estimate of GEBVs was considered as 0.95, rather than 1.00, to account for genotyping error.

#### Breeding strategies

The breeding strategies A_*p*_, A_*p*_W_*gs*_ and A_*gs*_W_*gs*_ were simulated in QuLinePlus using the modelling options available for each method.

- *Among half-sib family phenotypic selection* (A_*p*_): Using Sim200 and Sim1000 training populations, the A_*p*_ strategy was performed by randomly intermating all individuals to generate 196 and 980 HS families. These HS families were evaluated for DM yield in two environments for two years using three replicates and 30 plants per plot. From these trials, among family selection pressures of 20% and 2% were applied to select the best families. To restore the initial number of parents in the training population (196 and 980) for the next selection cycle, random samples of 5 (2.77%) and 50 (27.77%) individuals from the best 20% and 2% HS families were taken and used as parents to generate progeny for the next selection cycle. Each selection cycle was completed in three years (years 1 and 2—field evaluation and selection, and year 3—random mating of selected parents and half-sib family generation).
- *Among half-sib family phenotypic selection and within family genomic selection (*A_*p*_W_*gs*_*)*: In this breeding strategy, HS families were phenotypically evaluated as described in (a) and among family selection pressures of 20% and 2% were applied based on phenotype. However, within family selection was based on GEBVs estimated using the rrBLUP genomic prediction model. The GEBVs were ranked and the top 5 (2.77% within family selection pressure) or 50 (27.77% within family selection pressure) individuals from the best 20% and 2% families, respectively, were selected as parents for the next cycle. Each selection cycle was completed in three years (years one and two—field evaluation and selection, and year three—random mating of selected parents and half-sib family generation).
- *Among and within half-sib family selection based on genomic selection (A*_*gs*_*W*_*gs*_*)*: The A_*gs*_W_*gs*_ strategy was similar to A_*p*_W_*gs*_, with the only difference at the stage of among family selection, which was based on GEBVs rather than phenotypic measurements. Among and within family selection pressures were the same as the A_*p*_W_*gs*_ strategy, with 20% and 2% among family selection pressure and 2.77% and 27.77% within family selection pressure. It is important to note that this breeding strategy was conducted in one year.

#### Simulation output

Percentage genetic gain (%∆G) for all three breeding strategies was based on BLUP mean differences between selection cycles. The percentage genetic gain per year was calculated by dividing total predicted %∆G by the number of years per cycle. Cumulative genetic gain (Σ∆G) was calculated as the BLUP mean in each cycle relative to that in cycle zero. Allele fixation rate was computed within the QuLinePlus software to determine the percentage of fixed alleles in each cycle for the trait under selection. Genomic prediction accuracy was computed as the Pearson correlation coefficient between the true breeding value and their GEBVs. Percentage change of genetic variance across selection cycles was computed relative to cycle zero, considered as 100%.

## Results

### Deterministic modelling

Variance component analysis indicated significant (P < 0.05) additive genetic variation (${\sigma }_{A}^{2}$) for herbage dry matter (DM) yield among the HS families within each of the two mock data matrices, consisting of 200 and 1000 entries (Table 1). There were significant (P < 0.05) family-by-season (${\sigma }_{AS}^{2}$) and family-by-location (${\sigma }_{AL}^{2}$) interactions estimated from the 200 HS matrix but family-by-year (${\sigma }_{AY}^{2}$) was not significant (P > 0.05) in this dataset. The estimates of family-by-season (${\sigma }_{AS}^{2}$), family-by-location (${\sigma }_{AL}^{2}$) and family-by-year (${\sigma }_{AY}^{2}$) interactions were all significant (P < 0.05) for the 1000 HS family DM yield matrix. HS family narrow sense heritability (${h}_{n}^{2}$) estimates for DM yield, based on family mean performance across years, seasons and locations, were moderate for both data matrices, the 1000 HS family derived estimate being higher (Table 1). The estimates were within the range reported for perennial ryegrass^24,39,40^. The estimated genetic parameters, from REML analysis, presented in Table 1 were used as starting points in the different breeding equations to predict genetic gain.

**Table 1:** Ranges, medians, means (based on BLUP estimates), variance components and associated standard error (± SE) and family mean narrow sense heritability for herbage DM yield estimated from the 200 and 1000 HS family data; evaluated across two sites over 2 years and 3 seasons per year.

Sources of variation	200 HS	1000 HS
Range (kg ha^−1^)	2649–3662	2587–4073
Median (kg ha^−1^)	3298	3086
Mean (kg ha^−1^)	3262	3301
${\sigma }_{A}^{2}$	14,170 ± 5310	22,345 ± 3692
${\sigma }_{AY}^{2}$	2222 ± 1481	4102 ± 608
${\sigma }_{AS}^{2}$	13,406 ± 2292	12,942 ± 865
${\sigma }_{AL}^{2}$	38,511 ± 5107	58,532 ± 3420
${\sigma }_{\varepsilon }^{2}$	221,768 ± 4307	159,453 ± 1330
${h}_{n}^{2}$	0.310 ± 0.125	0.358 ± 0.043

The estimated variance components were used as starting points for simulation of breeding methods.

#### Predicted genetic gain based on the 200 HS family mock data matrix

Applying the among HS family phenotypic selection method (A_*p*_), at a selection pressure of 20%, to the seasonal DM yield data, predicted a 1.43% increase in DM yield above the population mean of 200 HS families (Table 2). Using phenomics with an accuracy of 0.90 for estimating DM yield generated from A_*Ph*_, a 1.29% increase in DM yield was predicted. The cost per %ΔGc using A_*p*_ was higher than that when A_*Ph*_ was applied. However, using A_*Ph*_ reduced the cost per %ΔGc by 25%. The final number of selected parents decreased from 40 at 20% selection pressure to 4 individuals at 2% selection pressure (Table 2).

**Table 2:** Simulated predictions of percentage genetic gain per cycle (3 years) of selection (%ΔGc), at different selection pressure (%), for perennial ryegrass dry matter (DM) yield and associated cost per percentage genetic gain ($ per % gain), from half-sib (HS) family phenotypic selection among 200 families evaluated across 2 years, 3 seasons and 2 locations.

Selection pressure (%)	A_*p*_ %ΔGc	A_Ph*_ %ΔGc	A_*p*_ $/% gain	A_*Ph*_ $/% gain	No. parents generated
20	1.43	1.29	102,739	76,987	40
10	1.80	1.62	81,724	61,239	20
5	2.11	1.90	69,822	52,321	10
2	2.47	2.22	59,436	44,714	4

A_*p*,_ DM yield data collected using seasonal herbage cuts; A_*Ph*_, DM yield data generated using seasonal phenomics based phenotyping. The costs associated with achieving one percent genetic gain based on DM cuts and phenomics were $7.50 and $0.87 per sample, respectively. The associated number of selected parents are indicated. A cycle of selection is the sum of the number of years of the field trial (2 years) plus one extra year for progeny generation, total 3 years. $, New Zealand dollars. These results are based on the estimated variance components, from REML analysis of the mock data, used as starting points for simulation of breeding methods.

Ph*, LiDAR based phenomics accuracy = 0.90.

The results of predicted ∆G presented in Tables 3 and 4 were generated from two cycles of HS family selection for increasing herbage DM yield. Cycle 1 (C1) (Table 3) was based on the A_*p*_W_*gs*_ method in which the same among and within family selection pressures of 20%/10% (among/within family), 10%/5%, 5%/1% and 2%/1%, were applied at each level of accuracy r_A_ (0.26, 0.36, 0.46). Cycle 1 consisted of 3 years. Cycle 2 (C2) (Table 4) used genomic selection for both the among and within family selection (A_*gs*_W_*gs*_) applying selection pressures of 20%/10% and 10%/5%. Cycle 2, a single year, was based on 100 HS families each taken from the 2000 HS and 500 HS families generated in C1 at selection pressures of 20%/10% and 10%/5%, respectively. In addition to r_A_ values of 0.26, 0.36 and 0.46, an additional level of accuracy, r_A_ of 0.12, was applied in C2 to model a scenario of declining accuracy due to decay of genetic relationships (Table 4).

**Table 3:** Simulation cycle 1 (C1) of selection for seasonal DM yield among 200 HS families of perennial ryegrass based on among HS family phenotypic selection, using data from a 2 year field trial, 3 seasons per year across 2 locations followed by the application of genomic selection (GS) within the selected HS families.

r_A_ C1	Selection pressure (*among/within*) (%) in C1	A_*p*_W_*gs*_ %ΔGc	A_*p*_W_*gs*_ ΔGc in absolute units (kg ha^−1^)	A_Ph_*W_*gs*_ %ΔGc	A_*Ph*_W_*gs*_ ΔGc in absolute units (kg ha^−1^)	A_*p*_W_*gs*_ $/% gain	A_*Ph*_W_*gs*_ $/% gain	No. parents generated in C1
0.26	20/10	2.88	93.95	2.59	84.55	111,819	105,778	400
0.36		3.44	112.21	3.10	100.99	93,703	88,375	
0.46		3.99	130.15	3.59	117.14	80,639	76,313	
0.26	10/5	3.49	113.84	3.14	102.46	68,655	61,135	100
0.36		4.15	135.37	3.74	121.84	57,865	51,327	
0.46		4.79	156.58	4.31	140.92	50,006	44,539	
0.26	5/1	4.3	140.27	3.87	126.24	46,218	39,009	10
0.36		5.14	167.67	4.63	150.90	38,635	32,606	
0.46		5.99	195.39	5.39	175.85	33,190	28,008	
0.26	2/1	4.67	152.34	4.20	137.10	37,303	30,087	4
0.36		5.51	179.74	4.96	161.76	31,592	25,477	
0.46		6.35	207.14	5.72	186.42	27,397	22,092	

Combinations of among and within (*among/within*) HS family selection at different selection pressures (%) and different genomic prediction accuracies (r_A_) were applied. The costs per percent genetic gain, relative to the mean BLUP value (3262 kg ha^−1^), of the 200 HS families, based on DM cuts and phenomics (*Ph*) were $7.50 and $0.87 per sample, respectively. For GS, $41 was used as the cost for generating a single GEBV (genomic estimated breeding value). A cycle of selection is the sum of the number of years of the field trial (2 years) plus one extra for progeny generation, total 3 years. Percentage genetic gain per cycle of selection (%ΔGc), cost per percentage of predicted genetic gain ($ per % gain), among Ap family phenotypic selection and within HS_*gs*_ family genomic selection (A_*p*_W_*gs*_) and *Ph* selection (A_*Ph*_W_*gs*_). r_A,_ is the Pearson correlation coefficient between Phenotypic Estimated Breeding Values and Genomic Estimated Breeding Values. $, New Zealand dollars. These results are based on the estimated variance components, from REML analysis of the mock data, used as starting points for simulation of breeding methods.

*LiDAR based phenomics accuracy = 0.90.

**Table 4:** Cycle 2 (C2) is based on selection among and within HS family progeny generated from polycrossing the elite parents identified in cycle 1.

Progeny**mean (kg ha^−1^) from C1 using A_*p*_W_*gs*_	r_A_ C2	Selection pressure (%) in C2	A_*gs*_W_*gs*_ %ΔGc	A_*gs*_W_*gs*_ ΔGc in absolute units (kg ha^−1^)	Total† %ΔG (C1 + C2)	Cost ($)† per %ΔG (C1 + C2)	No. parents generated in C2
3356	0.26	20/10	2.05	68.84	4.93	148,418	200
	*0.12*		*0.95*	*32.04*	*3.83*	*191,044*	
3374	0.36		2.82	95.32	6.26	116,885	
	*0.12*		*0.95*	*32.04*	*4.39*	*166,674*	
3392	0.46		3.59	121.79	7.58	96,530	
	*0.12*		*0.94*	*32.04*	*4.93*	*148,418*	
3376	0.26	10/5	2.44	82.45	5.93	109,562	50
	*0.12*		*1.14*	*38.37*	*4.63*	*140,324*	
3397	0.36		3.36	114.16	7.51	86,511	
	*0.12*		*1.13*	*38.37*	*5.28*	*123,049*	
3419	0.46		4.27	145.87	9.06	71,711	
	*0.12*		*1.12*	*38.37*	*5.91*	*109,932*	

In C2, both among and within HS selection is based only on genomic selection (GS). Combinations of among and within (*among/within*) HS family selection at selection pressures of among/within, 20%/10% and 10%/5%, and different genomic prediction accuracies (r_A_) were applied. From within each of 100 HS families generated from cycle 1 generated at 20%/10% and 10%/5% selection pressure, random samples of 100 seedlings per family were used in cycle 2, a total of 10,000 individuals subjected to GS. The costs per percent genetic gain (relative to the C1 HS family predicted progeny means), are based on $41 for generating a single GEBV (genomic estimated breeding value). The length of C2 is 1 year. percentage genetic gain per cycle of selection (%ΔGc), cost per percentage of predicted genetic gain ($ per % gain). r_A,_ is the Pearson correlation coefficient between Phenotypic Estimated Breeding Values and GEBV’s, based on the predicted equation developed in C1. Among and within half-sib family genomic selection (A_*gs*_W_*gs*_). In C2 an r_A_ of 0.12, predicted in cycle 2 of stochastic simulation using Sim200 HS families, was used in deterministic simulation to model a possible scenario of r_A_ decay, displayed in italics in the table.

**, mean of 200 HS families plus predicted ΔG in C1; †, sum of gain (%ΔGc) and cost ($) from C1and C2.

In C1 (Table 3), the predicted %ΔG per cycle rose as r_A_ was increased. In this cycle, the combination of increasing among HS family phenotypic selection pressure (2%) and within family genomic selection pressure (1%), at high r_A_ (0.46), resulted in the highest ΔG of 6.35%, based on data from the A_*p*_ seasonal herbage DM yield sampling method. The cost per percentage gain was a low $27,397 compared to the high $111,819 at among and within HS family selection pressures of 20% and 10%, respectively, at r_A_ of 0.26. However, while the low selection pressures and r_A_ resulted in the selection of 400 parents, the high selection pressure and r_A_ lead to identifying only 4 parents (Table 3). As expected, the predicted ΔG per cycle, under the A_*Ph*_ strategy phenomics assessments of seasonal herbage DM in C1, was slightly lower than A_*p*_, but there was a substantial increase in cost efficiency per % genetic gain (Table 3).

Cycle 2 (Table 4) was based on genomic selection among and within (A_*gs*_W_*gs*_) 100 HS families generated from selected parents from C1 at 20%/10% and 10%/5% selection pressure and at r_A_ values of 0.26, 0.36 and 0.46. In C2, ΔG was estimated relative to the new mean of HS progeny generated from C1, within each combination of % selection pressure and r_A_. The predicted ΔG per cycle ranged from 2.05% at r_A_ 0.26 to 3.59% at r_A_ 0.46, at among and within family selection pressures of 20%/10%. Applying higher selection pressures (10%/5%) at r_A_ values of 0.26 and 0.46, increased the range of predicted ΔG per cycle to 2.44% and 4.27%, respectively. Predicted ΔG combined across selection C1 and C2 resulted in totals ranging from 4.93% to 7.58% at r_A_ 0.26 and 0.46, respectively, at among and within selection pressures of 20%/10% (Table 4). At the same r_A_ values of 0.26 and 0.46 at among and within HS family selection pressures of 10%/5%, the predicted ΔG combined across cycles C1 and C2 were 5.93% and 9.06%, respectively. Comparing the costs per percentage predicted ΔG, combined across cycles C1 and C2, the total of $148,418 at r_A_ 0.26 and 20%/10% among and within family selection pressures was over double the cost, $71,711 at r_A_ 0.46 at 10%/5% selection pressures. The number of elite parents selected on GEBV’s at the end of C2 were 200 and 50 based on the among and within family selection pressures of 20%/10% and 10%/5%, respectively (Table 4). These results assumed that the r_A_ values, 0.26, 0.36 and 0.46, did not change from C1 to C2. The effect of possible genomic accuracy decay on ΔG in C2 was assessed using a r_A_ of 0.12. This resulted in diminished ΔG and increased costs ($) per % ΔG. However, even at the reduced r_A_ of 0.12, the additional predicted ΔG in the single year of C2 provided an increase in total gain across cycles C1 and C2.

#### Predicted genetic gain based on the 1000 HS family mock data matrix

The trend of response to selection for DM yield based on the 1000 HS families was the same as that observed from the 200 HS family simulations. Using DM cut phenotypic data (A_*p*_) the predicted ΔGc ranged from 1.90 at 20% among HS family phenotypic selection pressure to 3.62% at 1% selection pressure (Table 5). The large population of HS families resulted in higher numbers of parental half-sibs being selected, compared to the 200 HS family dataset. This ranged from 200 parental HS families at 20% selection pressure to 10 families at 1%. The application of phenomics based DM assessment (A_*Ph*_) clearly indicated its impact on reducing costs ($) per %ΔGc by 58%.

**Table 5:** Simulated predictions of percentage genetic gain per cycle (3 years) of selection (%ΔGc) and cost per percentage of predicted genetic gain ($ per % gain), using among half-sib (A_*p*_) family selection, for dry matter (DM) yield based on the 1000 HS family data (evaluated across years, seasons and locations), from DM cuts (A_*p*_) and phenomics based phenotyping (A_*Ph*_).

Selection pressure (%)	A_*p*_ %ΔGc	A_*Ph*_ %ΔGc	A_*p*_ $/% gain	A_*Ph*_ $/% gain	No. parents generated
20	1.90	1.71	203,158	86,152	200
10	2.38	2.14	162,185	68,777	100
5	2.79	2.51	138,351	58,670	50
2	3.28	2.95	117,683	49,905	20
1	3.62	3.26	106,630	45,218	10

Selection pressure (%) and the associated number of selected parents are indicated. $, New Zealand dollars. These results are based on the estimated variance components from REML analysis, used as starting points for simulation of breeding methods.

Ph*, LiDAR based phenomics accuracy = 0.90.

Analyses involving 1000 HS families (Tables 6 and 7) generated results analogous to those from the 200 HS families (Tables 3 and 4). The application of the A_*p*_W_*gs*_ breeding strategy to the 1000 HS families in C1 resulted in predicted ΔGc ranging from 3.69% to 8.10% at among/within selection pressures of 20%/10% and 2%/1% at r_A_ values of 0.26 and 0.46, respectively. While the predicted ΔGc from applying A_*Ph*_W_*gs*_ at similar combinations of selection pressures and r_A_ values was lower, there was a considerable reduction in cost ($) per %ΔG that ranged from 10 to 40%. The number of selected parental HS families in C1 at the among and within family selection pressure combinations of 5%/1% and 2%/1%, resulted in selection of less than 100 HS families, 50 and 20, respectively (Table 6).

**Table 6:** Simulation cycle 1 (C1) of selection for seasonal DM yield among 1000 HS families of perennial ryegrass based on among family phenotypic selection, using data from a field trial conducted across 2 locations, 2 years with 3 seasonal measurements per year.

r_A_ C1	Selection pressure (*among/within*) (%) in C1	A_*p*_W_*gs*_ %ΔGc	A_*p*_W_*gs*_ ΔGc in absolute units (kg ha^−1^)	A_Ph_*W_*gs*_ %ΔGc	A_*Ph*_W_*gs*_ ΔGc in absolute units (kg ha^−1^)	A_*p*_W_*gs*_ $/%/gain	A_*Ph*_W_*gs*_ $/%/gain	No. parents generated in C1
0.26	20/10	3.69	121.85	3.32	109.67	338,618	304,372	2000
0.36		4.38	144.64	3.94	130.17	285,274	256,423	
0.46		5.07	167.42	4.56	150.68	246,450	221,525	
0.26	10/5	4.49	148.05	4.04	133.25	186,971	148,681	500
0.36		5.29	174.72	4.76	157.25	158,696	126,196	
0.46		6.10	201.39	5.49	181.25	137,623	109,439	
0.26	5/1	5.51	182.00	4.96	163.80	115,154	79,819	50
0.36		6.56	216.56	5.90	194.91	96,723	67,043	
0.46		7.61	251.13	6.85	226.02	83,377	57,792	
0.26	2/1	6.00	198.10	5.40	178.29	85,250	50,522	20
0.36		7.05	232.66	6.35	209.40	72,553	42,998	
0.46		8.10	267.23	7.29	240.51	63,148	37,424	

Genomic selection (GS) was applied to individuals within the selected HS families. Combinations of among and within (*among/within*) HS family selection at different selection pressures (%) and different genomic prediction accuracies (r_A_) were applied. The costs per percent genetic gain, relative to the mean BLUP value (3301 kg ha^-1^), of the 1000 HS families, based on DM cuts and phenomics (*Ph*) were $7.50 and $0.87 per sample, respectively. For GS, $41 was used as the cost for generating a single GEBV (genomic estimated breeding value). A cycle of selection is the number of years of the field trial (2 years) plus one extra for progeny generation, total 3 years. Percentage genetic gain per cycle of selection (%ΔGc), cost per percentage of predicted genetic gain ($ per % gain), among Ap family phenotypic selection and within HS_*gs*_ family genomic selection (A_*p*_W_*gs*_) and *Ph* selection (A_*Ph*_W_*gs*_). r_A,_ is the Pearson correlation coefficient between Phenotypic Estimated Breeding Values and Genomic Estimated Breeding Values. $, New Zealand dollars. These results are based on the estimated variance components from REML analysis, used as starting points for simulation of breeding methods.

Ph*, LiDAR based phenomics accuracy = 0.90.

**Table 7:** Cycle 2 (C2) is based on genomic selection (GS) applied to among and within HS family progeny generated from polycrossing the elite parents identified in cycle 1.

Progeny**mean (kgha^−1^) from C1 using A_*p*_W_*gs*_	r_A_ C2	Selection Pressure (%) in C2	A_*gs*_W_*gs*_ %ΔGc	A_*gs*_W_*gs*_ ΔGc in absolute units (kg ha^−1^)	Total† %ΔG (C1 + C2)	Cost ($)† %ΔG (C1 + C2)	No. parents generated in C2
3423	0.26	20/10	2.53	86.44	6.22	273,392	200
	*0.12*		*1.17*	*39.90*	*4.86*	*349,897*	
3446	0.36		3.47	119.69	7.85	216,624	
	*0.12*		*1.16*	*39.90*	*5.54*	*306,949*	
3468	0.46		4.41	152.94	9.48	179,378	
	*0.12*		*1.15*	*39.90*	*6.22*	*273,392*	
3449	0.26	10/5	3.00	103.54	7.49	172,296	50
	*0.12*		*1.39*	*47.79*	*5.88*	*219,473*	
3476	0.36		4.12	143.36	9.41	137,141	
	*0.12*		*1.37*	*47.79*	*6.66*	*193,769*	
3502	0.46		5.23	183.18	11.33	113,901	
	*0.12*		*1.36*	*47.79*	*7.46*	*172,989*	

Combinations of among and within (*among/within*) HS family selection at pressures of among/within, 20%/10% and 10%5%, and different genomic prediction accuracies (r_A_) were applied. From within each of 100 HS families from cycle 1 generated at 20%/10% and 10%/5% selection pressure, random samples of 100 seedlings per family were used in cycle 2, a total of 10,000 individuals subjected to GS. The costs per percent genetic gain (relative to the C1 HS family predicted progeny means), are based on $41 for generating a single GEBV (genomic estimated breeding value). The length of C2 is 1 year. Percentage genetic gain per cycle of selection (%ΔGc), cost per percentage of predicted genetic gain ($ per % gain). r_A,_ is the Pearson correlation coefficient between Phenotypic Estimated Breeding Values and Genomic Estimated Breeding Values, based on the predicted equation developed in C1. Among and within half-sib family genomic selection (A_*gs*_W_*gs*_). In C2 a r_A_ of 0.12, predicted in cycle 2 of stochastic simulation using Sim200 HS families, was used in deterministic simulation to model a possible scenario of r_A_ decay, displayed in italics in the table.

**, mean of 1000 HS families plus predicted ΔG in C1; †, sum of gain (%ΔGc) and costs ($) from C1and C2.

Genomic among and within family selection was conducted in C2, in a single year, on 100 HS families from C1 generated at 20%/10% and 10%/5% selection pressure at r_A_ values of 0.26, 0.36 0.46. Trends in ΔGc mirrored those observed with the 200 HS family dataset, but the level of gain was consistently higher. The A_*gs*_W_*gs*_ applied resulted in predicted ΔGc ranging from 2.53% at r_A_ 0.26 to 4.41% at r_A_ 0.46 at among and within family selection pressures of 20%/10% (Table 7). At selection pressures of 10%/5% and r_A_ values of 0.26 and 0.46, predicted ΔGc cycle increased to 3.00% and 5.23%, respectively. Combining %ΔG across C1 and C2 resulted in total ΔG ranging from 6.22% to 9.48% at r_A_ 0.26 and 0.46, respectively, at selection pressures of 20%/10%. At the same r_A_ values of 0.26 and 0.46 and selection pressures of 10%/5%, the predicted %ΔG combined across selection C1 and C2 was 7.49% and 11.33%, respectively. The total combined costs per %ΔG across cycles 1 and 2, were $273,392 at r_A_ equal to 0.26 at 20%/10% among and within family selection pressures was over twice the cost, $113,901 at r_A_ 0.46 at 10%/5% selection pressures. The elite parents selected on GEBV’s at the end of cycle 2 were 200 individuals and 50 individuals based on the selection pressures of 20%/10% and 10%/5%, respectively (Table 7). As in the 200 HS family analysis, the effect of a possible scenario of r_A_ decay on ΔG in C2 was assessed using a r_A_ of 0.12, (Table 7). The outcomes were similar to those observed in the 200 HS analysis.

**Figure 1:**
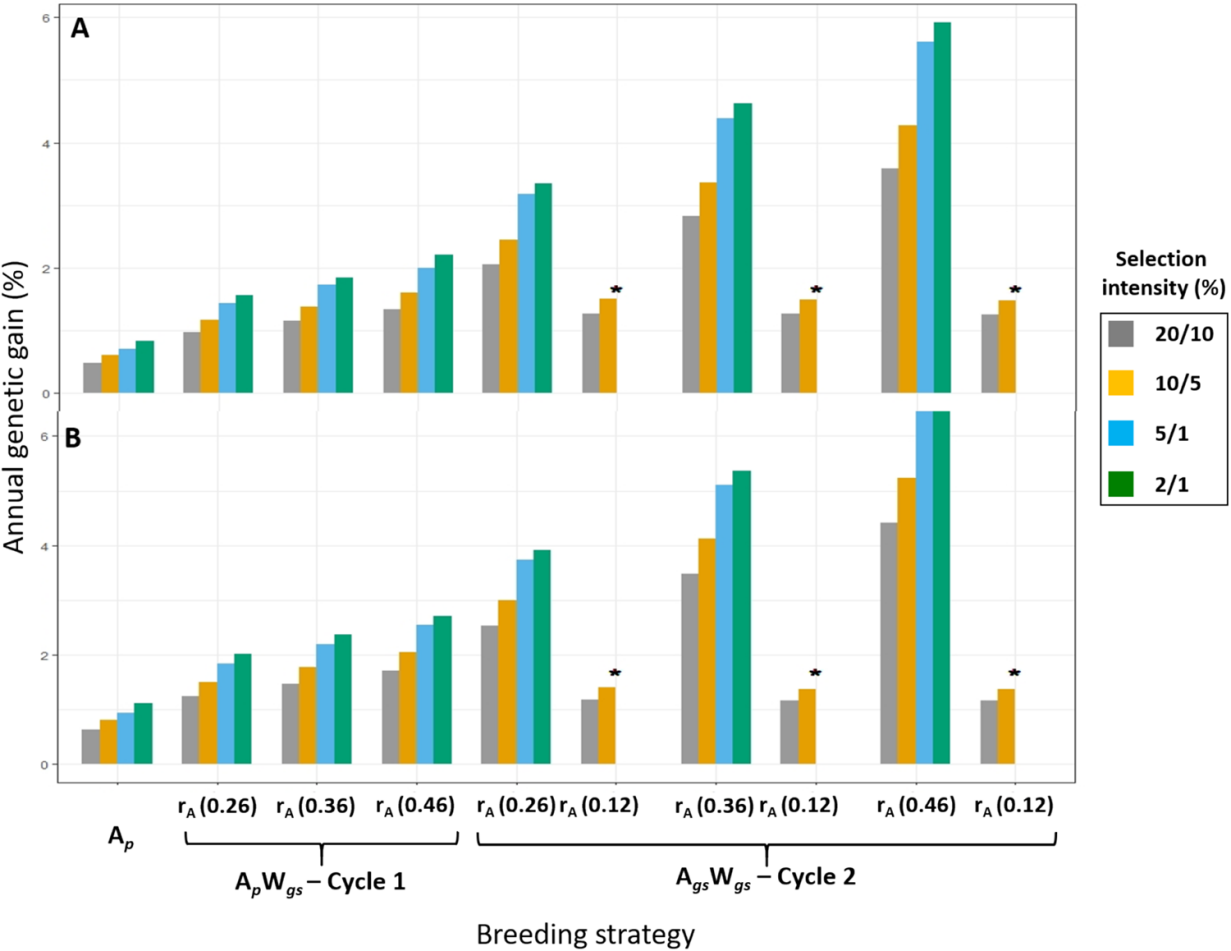
Predicted annual percentage genetic gain (%∆G) for seasonal DM yield resulting from modelling the breeding strategies; among HS family phenotypic selection (A_*p*_), among HS family phenotypic selection and within family genomic selection (A_*p*_W_*gs*_), among and within HS family genomic selection (A_*gs*_W_*gs*_), applied to the: (**A**) 200 HS families and (**B**) 1000 HS families, of perennial ryegrass. A_*p*_ was only conducted for one cycle. In cycle 2, only genomic selection (GS) was conducted at accuracies (r_A_) of 0.12, 0.26, 0.36 and 0.46. *r_A_ of 0.12 was only applied to simulations using among HS family selection pressures of 20% and 10% and within HS family selection pressures of 10% and 5%, respectively. The predicted annual %∆G for each combination of selection pressures is indicated by the height of bars, values in brackets indicate r_A_ levels. Since cycle 1 consisted of 3 years, annual genetic gain, %∆G = %∆G_C_/3. Cycle 2 was 1 year.

Predicted annual %ΔG for seasonal DM yield resulting from deterministic modelling of the three breeding strategies; A_*p*_, A_*p*_W_*gs*_ and A_*gs*_W_*gs*_, based on data from the 200 and 1000 HS families clearly indicate the lower response to selection resulting from the among HS family phenotypic selection (A_*p*_) breeding strategy (Fig. 1A,B). There was an increase in annual %ΔG when GS was combined with the A_*p*_ breeding method. The predicted annual %ΔG improved with increasing selection pressure and r_A_. The breeding strategy A_*gs*_W_*gs*_ resulted in the highest predicted annual %ΔG at all among and within family selection pressures and r_A_. It is also clear that when r_A_ decreased to 0.12 in C2 there was a reduction in predicted annual %ΔG, as would be expected. However, even at a r_A_ value of 0.12 in C2, the predicted annual %ΔG was higher than that predicted for HS in C1 at all selection pressures (Fig. 1A,B).

### Stochastic modelling

#### Genetic gain

**Figure 2:**
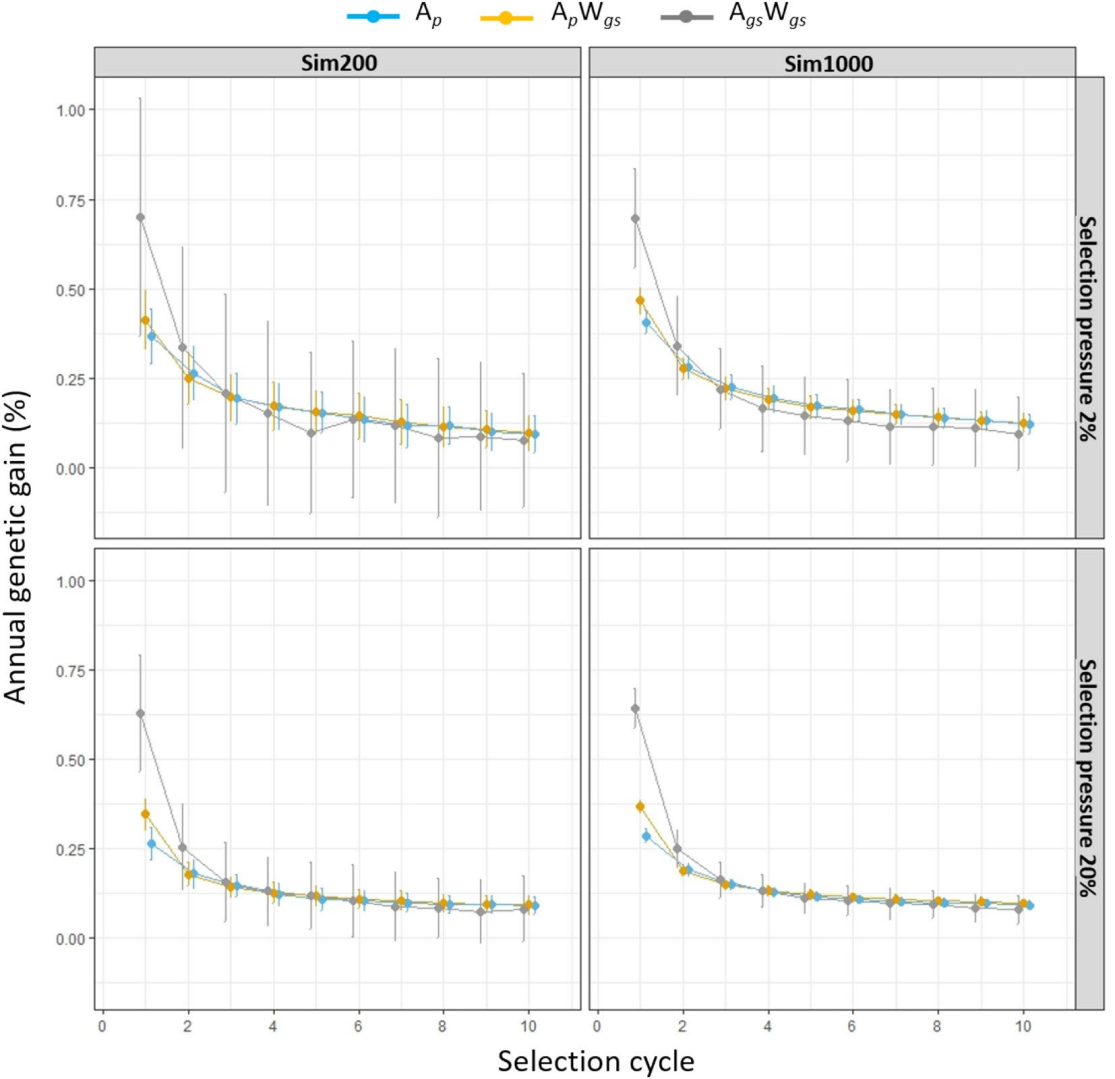
The percentage genetic gain (%ΔG) per year for DM yield in Sim200 and Sim1000 training populations estimated across 10 selection cycles using three breeding strategies (A_*p*_, A_*p*_W_*gs*_ and A_*gs*_W_*gs*_). Selection pressures of 20% and 2% were imposed to select the best HS families. Within each family, for the A_*p*_W_*gs*_ and A_*gs*_W_*gs*_ strategies the top 5 or 50 individuals were selected and for the A_*p*_ strategy 5 or 50 individuals were randomly selected to restore the initial number of parents for the next selection cycle. In each selection cycle %ΔG was based on 250 iterations.

The percentage annual genetic gain (%ΔG) for DM yield in Sim200 and Sim1000 training populations were different for each breeding strategy (Fig. 2). The trends in genetic gain between two training populations (Sim200 and Sim1000) were similar, however, the variation in genetic gain across multiple iterations was higher in Sim200 compared to the Sim1000 training population. Among different breeding strategies, the highest annual genetic gain (%ΔG) was achieved for A_*gs*_WS_*gs*_, followed by A_*p*_W_*gs*_ and A_*p*_ strategies under both 20% and 2% selection pressures. The differences in %ΔG between the breeding strategies were less evident after the second selection cycle (Fig. 2). When comparing among family selection pressures, the highest genetic gain for all three strategies was observed under 2% selection pressure compared to 20% selection pressure. While the variability in genetic gain across multiple iterations was consistently higher under 2% selection pressure compared to that at 20%, this was more evident for the A_*gs*_W_*gs*_ strategy. The highest ΣΔG in each selection cycle was achieved in A_*p*_W_*gs*_ strategy, followed by A_*p*_ and A_*gs*_W_*gs*_ (Supplementary material 1, Figure S2).

#### Fixation rate of favourable alleles

**Figure 3:**
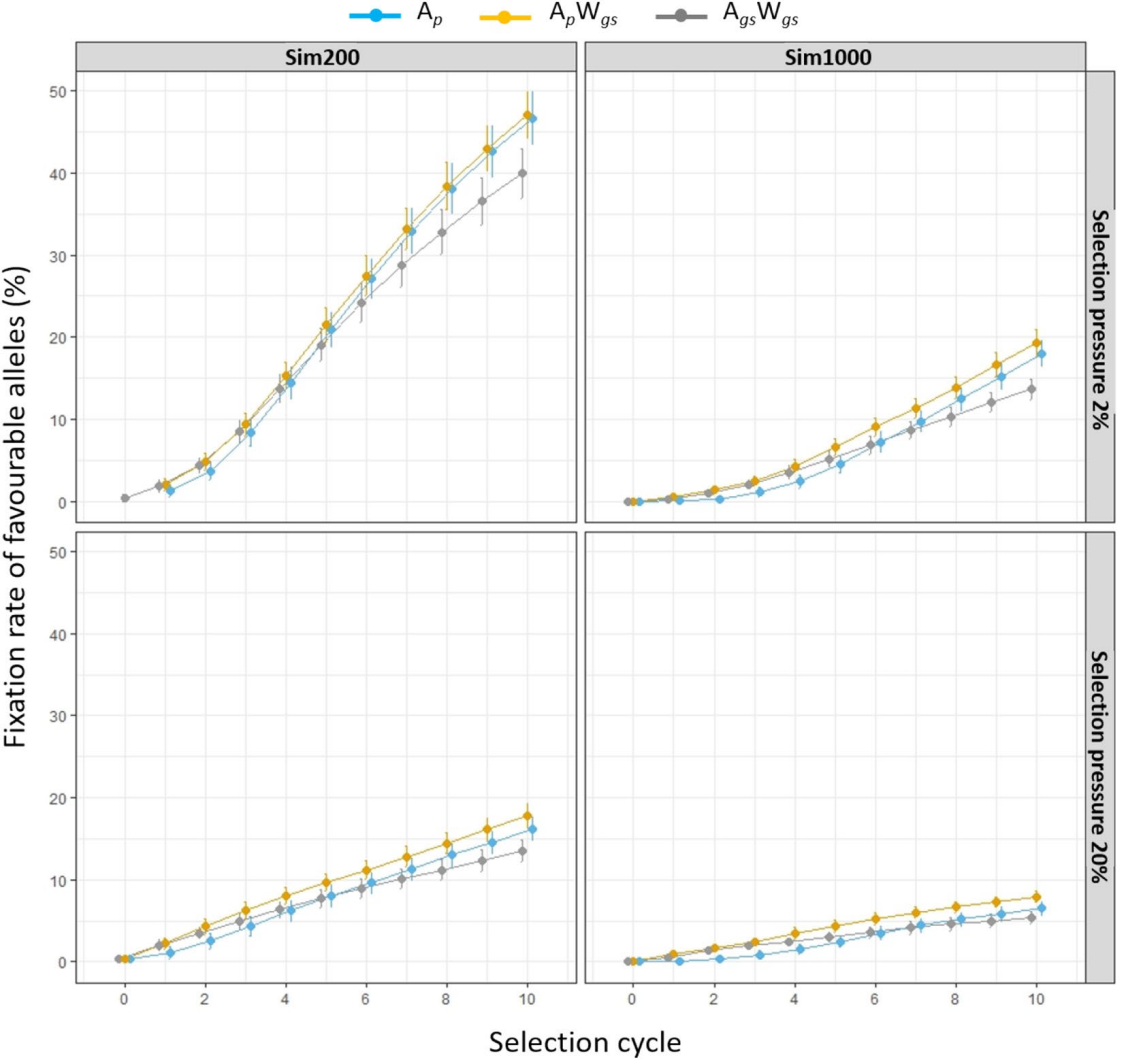
Percentage of favourable alleles fixed in the Sim200 and Sim1000 training populations at each selection cycle, under three breeding strategies (A_*p*_, A_*p*_W_*gs*_ and A_*gs*_W_*gs*_). A selection pressure of 20% and 2% was imposed to select the best HS families. Within each family, for the A_*p*_W_*gs*_ and A_*gs*_W_*gs*_ strategies the top 5 or 50 individuals were selected and for A_*p*_ strategy 5 or 50 individuals were randomly selected to restore the initial number of parents for next selection cycle. In each selection cycle percentage of favourable alleles were based on 250 iterations.

The influence of selection pressure and training population size on allele fixation rates were observed, with the latter being more pronounced (Fig. 3). The fixation rate was highest in the Sim200 training population under 2% selection pressure and the lowest rate was observed in Sim1000 under 20% selection pressure. In both the training populations, under 20% among family selection pressure, the fixation rate steadily increased across the selection cycles. However, under 2% selection pressure the alleles were fixed more rapidly from selection cycle 3 for all three breeding strategies. Among three breeding strategies, the allele fixation rate followed similar patterns and the differences in fixation rate were more noticeable in the later selection cycles (Fig. 3).

#### Genetic variance and prediction accuracy

**Figure 4:**
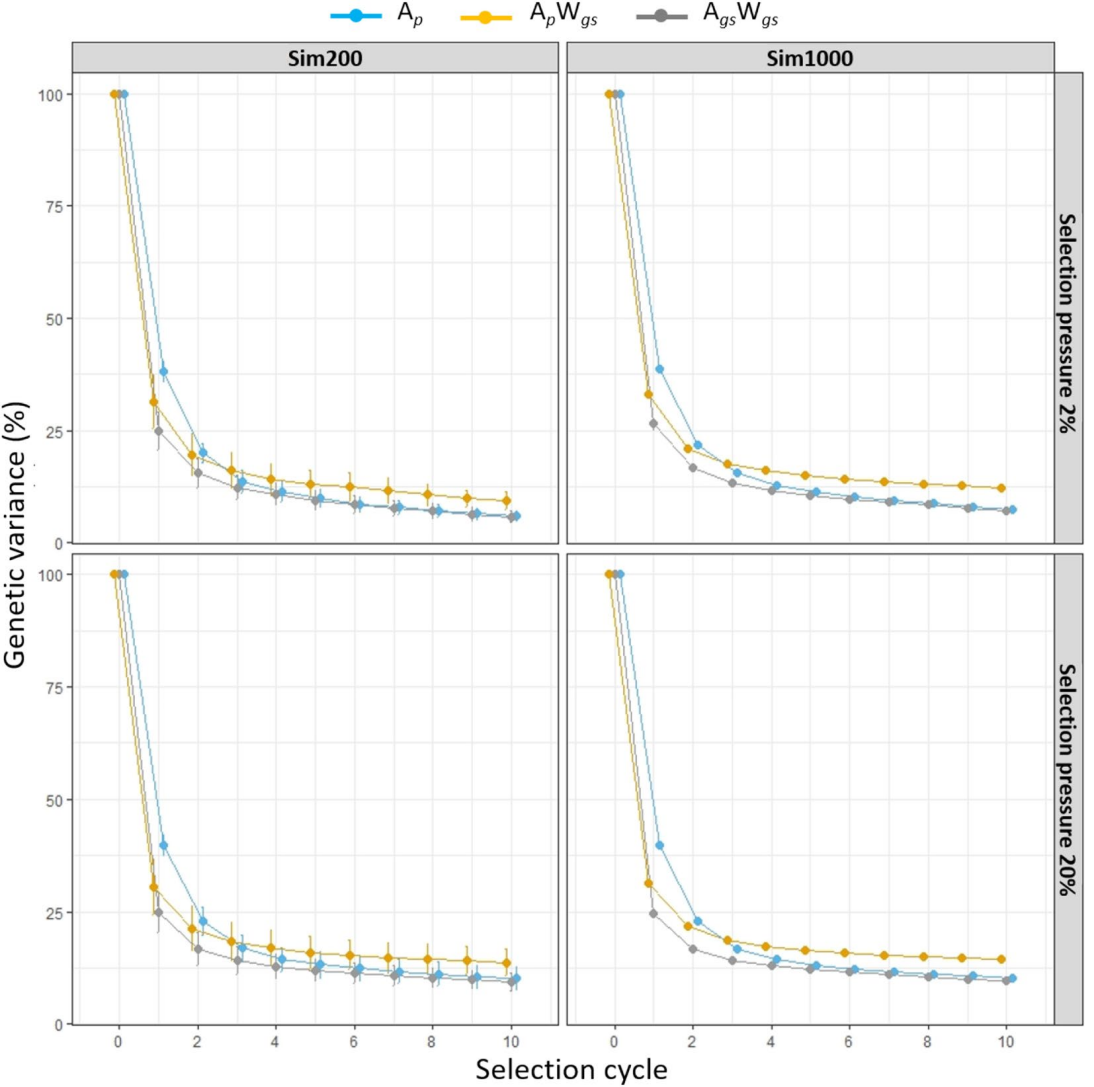
The percentage of genetic variance at each selection cycles estimated in Sim200 and Sim1000 training populations using three breeding strategies (A_*p*_, A_*p*_W_*gs*_ and A_*gs*_W_*gs*_). A selection pressure of 20% and 2% was imposed to select best half-sib families and within each family, for the A_*p*_W_*gs*_ and A_*gs*_W_*gs*_ strategies top 5 and 50 individuals were selected and for A_*p*_ strategy random 5 and 50 individuals were selected to restore the initial number of parents for next selection cycle. In each selection cycle percentage genetic variance was based on 250 iterations.

**Figure 5:**
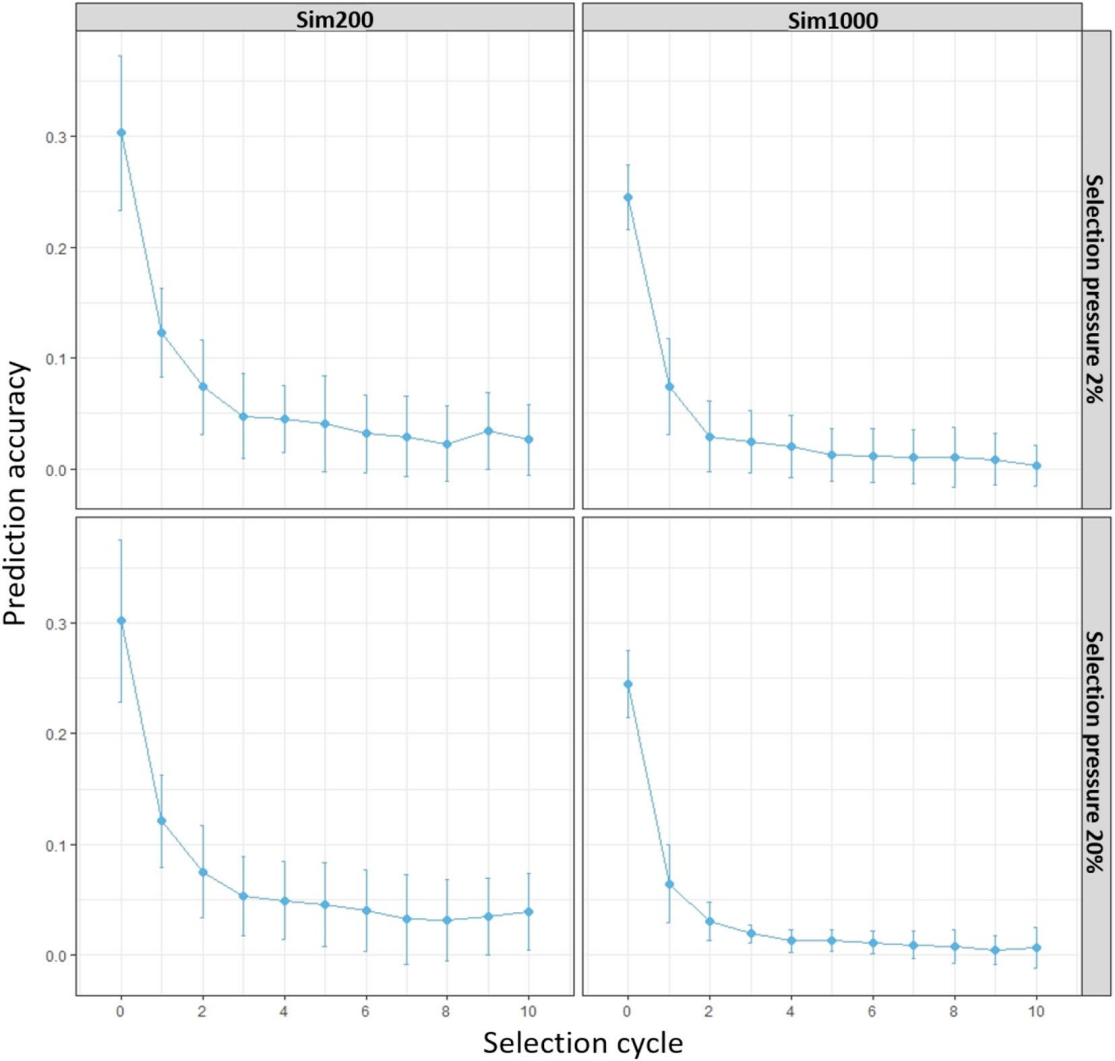
Average prediction accuracy estimated as the Pearson correlation co-efficient between true breeding value (TBV) and genomic estimated breeding value (GEBV) in the Sim200 and Sim1000 training populations. In each selection cycle prediction accuracy was based on 250 iterations.

The percentage of genetic variance present within the Sim200 and Sim1000 training population decayed rapidly from cycle 0 to cycle 1 for all three breeding strategies. The patterns were similar under the two different selection pressures (Fig. 4). In Sim200 training population, among three breeding strategies, the lowest genetic variance was observed for A_*gs*_W_*gs*_ followed by A_*p*_W_*gs*_ and A_*p*_, similar trends were observed in Sim1000 training population. The prediction accuracy at cycle 0 was 0.3 in Sim200 and 0.25 in Sim1000 and was reduced to 0.12 (60% decrease in prediction accuracy) and 0.07 (72% decrease in prediction accuracy) in selection cycle 1 (Fig. 5). There was no difference in the prediction accuracies between the two different selection pressures. The accuracy steadily decreased from cycle 2 and at the end of cycle 10 was a low 0.03 in Sim200 and 0.002 in Sim1000 training populations (Fig. 5).

## Discussion

### Deterministic modelling

Choosing and optimizing an appropriate breeding strategy to suit a specific cultivar development goal, will require the comparison of different breeding methods based on predicted genetic gains^3^. While a key criterion for determining the success of a breeding method is genetic gain, cost is also a major factor. Especially in commercial breeding programs, where cost efficiency of a breeding strategy, and access to commercial opportunities, will often determine its adoption.

With increasing pressure on plant breeders to develop new productive cultivars with adaptation to changing environments and meeting consumer demands, new selection and phenotyping technologies must be implemented to enhance genetic advance^41^. However, incorporation of these technologies into conventional field breeding programs are often challenged as being expensive to implement. Application of quantitative genetic deterministic^21^ and stochastic modelling^22^ can be used to evaluate the relative efficiency, genetic gain and associated cost, of conventional breeding strategies in combination with the integration of new selection technologies.

As mentioned in the introduction, HS and FS family breeding strategies are commonly used in forage grass cultivar development programs. In this paper, we present results from deterministic modelling using DeltaGen and stochastic modelling via QU-GENE/QuLinePlus, to predict ∆G and calculate cost per predicted %∆G for DM herbage yield of perennial ryegrass. Deterministic analysis was conducted using a common set of starting points based on estimates of quantitative genetic parameters, from analysis of the 200 HS and 1000 HS family mock data matrices generated using actual field trial DM yield (kg ha^-1^). We also evaluated the effect of using phenomics (*Ph*) on the cost per cycle of selection. The narrow sense heritability values 0.31 and 0.36, estimated for herbage DM yield for the 200 HS and 1000 HS families, respectively, were within the range previously reported^24,42,43^. For mean herbage DM yield across years, seasons and locations, and based on a three year selection cycle, the predicted annual %∆G per selection cycle from the A_*p*_ strategy varied from 0.48 to 0.83 (200 HS families) and 0.63 to 1.21 (1000 HS families), depending on selection pressure. These increases are in the general range reported by Humphreys^44^ 0.38%, Easton, et al.^45^ 0.4% to 0.5%, Wilkins and Humphreys^46^ 0.5% to 0.6%, Woodfield^47^ 0.25% to 0.73% and Harmer, et al.^48^ 0.76%. However, at high selection pressures applying A_*p*_ in large HS populations, such as the 1000 family dataset, annual increases of 1.21% can be achieved. With every percentage increase in ∆G, cost efficiency improved. Although the predicted %∆G using data from *Ph* based DM assessments (at an accuracy of 0.90), was lower than predictions using DM yield from herbage cuts, there was a considerable decrease in the cost per %∆G. This was especially evident when comparing DM measurement costs between the A_*p*_W_*gs*_ and A_*Ph*_W_*gs*_ breeding methods based on herbage cuts and LiDAR phenomic assessments, from the 1000 HS families. There was a considerable reduction in cost ($) per %ΔG that ranged from 10 to 40%.

Annual increases in %∆G of over 2% per year will be required in crops such as maize (*Zea mays*), rice (*Oryza sativa*), wheat (*Triticum aestivum*), and soybean (*Glycine max* L.), to meet future global food demands^49^. For perennial ryegrass and other out crossing forage species, achieving a 2% annual gain in DM yield using A_*p*_ methods, which exploit only a quarter of the total available additive genetic variation^27^, is an unrealistic objective. Accessing the ¾ additive variation within HS families will make a significant contribution to increasing annual %∆G^4,50^. Low genetic gains in forage breeding programs are due, in part, to an inability to satisfactorily exploit within family genetic variation^50,51^. Historically, application of within family selection in forage grasses could only be based on measurements conducted on random samples of individual, spaced plants, representing the selected HS families. Casler^50^ presented a range of examples in forage grass breeding for and against using spaced plants. Hayward and Vivero^52^ and Lazenby and Rogers^53^ indicated poor genetic correlation between individual spaced plant vigour and sward DM yield. Applying GS based on prediction models constructed using multi-year-season-location phenotypic data from sown rows or plots makes within family selection for DM yield and other sward traits feasible. The benefits to ∆G of using GS typically focus on the promise of reducing generation interval. However, in addition, the application of genomic prediction to generate GEBV’s for large numbers of random individuals sampled from elite HS families, offers a means to also improve ∆G by increasing the efficiency of within family selection for sward DM yield. Deterministic simulation of the A_*p*_W_*gs*_ breeding strategy using DeltaGen, based on the 200 HS and 1000 HS families, clearly indicated the advantage of applying GS for within family selection. For both HS populations, increasing within family selection pressure, from 10 to 1% or increasing r_A_ from 0.26 to 0.46 increased %∆G per cycle. For both the 200 HS and 1000 HS family populations, at 1% within family GS, %∆G per cycle for DM yield was 6.35 and 8.10, respectively. Based on the three-year selection cycle assumed in this study, these increases equate to annual %∆G of 2.11 and 2.70, comfortably above the 2% ∆G per annum target. As expected, with increasing genetic gain the cost %∆G decreased.

As alluded to above, in addition to enabling within family selection, a key advantage in using GS is reducing the length of a selection cycle and the cost %∆G^54,55^. This was further demonstrated in our deterministic simulation by applying a wholly GS second selection cycle, A_*gs*_W_*gs*_, to the HS families generated following C1 in both the 200 HS and 1000 HS family scenarios. The fact that A_*gs*_W_*gs*_ enabled selection of another generation of elite parental seedlings based on GEBV’s in the space of one year, following C1 (3 years), resulted in high annual %∆G in cycle 2. Annual %∆G resulting from A_*gs*_W_*gs*_ was higher than both the A_*p*_ and A_*p*_W_*gs*_ breeding strategies at all selection pressures and r_A_’s. This additional year based on A_*gs*_W_*gs*_ following the 3 years of the field-based strategy (C1) increased total %∆G.

It is important to note that the deterministic simulation results discussed assumed that the r_A_ values 0.26, 0.36 and 0.46 in C1 continued to hold in C2. There is always the probability of the correlation between GEBV’s and phenotype derived BLUP’s or true breeding values, decreasing due to decay in r_A_, due principally to declining genetic relatedness between training and selection populations^56^, following polycrossing of C1 selected parents. The use of a r_A_ of 0.12 was introduced into simulation in C2 to investigate this scenario. Simulation results indicated that even at the r_A_ value of 0.12 in C2, the predicted annual %ΔG using A_*gs*_W_*gs*_, was higher than that predicted for A_*p*_ in C1 at all selection pressures.

### Stochastic modelling

While genetic gain is a key determinant of the merit of a breeding strategy, information on fixation rate of favourable alleles and the rate of decay of genetic variance and r_A_ are vital, especially when designing long-term recurrent selection breeding programs. For example, knowing at what stage in the recurrent selection process to recalibrate the prediction model when using GS is essential. Application of strategic decision support software tools such as QU-GENE, provide a platform for plant breeders to assess the progress, over multiple selection cycles, for ∆G and associated genetic parameters such as; additive genetic variance, fixation rates and r_A_. In crop species, several studies have explored stochastic modelling to understand long-term trends of implementing GS in breeding programs^19,57–60^. In forages, Lin, et al.^55^ and Esfandyari, et al.^61^ used stochastic modelling to assess the impact of GS in commercial and FS breeding programs.

In the current study, stochastic modelling using QU-GENE/QuLinePlus provided graphical trends for; ∆G, allele fixation rate, genetic variance and r_A_, over 10 cycles of selection in response to three breeding strategies; A_*p*_, A_*p*_W_*gs*_ and A_*gs*_W_*gs*_, when applied to either 200 HS or 1000 HS families of perennial ryegrass. In our simulations, the greatest %∆G observed for GS (A_*p*_W_*gs*_ and A_*gs*_W_*gs*_) breeding strategies compared to A_*p*_ across two different selection pressures and populations sizes (Fig. 2). These results are due to precise selection of genotypes, for DM yield, within a family when implementing GS, compared to random selection of individuals from within selected families when using the A_*p*_ strategy. This further supports the findings of Esfandyari, et al.^61^ who in the context of full sib (FS) family breeding strategies, reported that accurate selection of single plants, based on GS, establishes a strong genetic correlation in terms of performance between single plants and plots, leading to increased ∆G.

The GS breeding strategies applied in C1 outperformed the A_*p*_ strategy in terms of predicted %∆G. However, in later cycles, these differences were less pronounced, due to a decline in r_A_ in the selection cycles post C1. These trends observed in our simulations were similar for both the Sim200 and Sim1000 HS families, mainly due to similar size and number of additive genetic effects used in the recombination map for simulating the breeding strategies. Generally, with larger training populations, r_A_ increases^6,62^ or remains constant relative to smaller populations^58^. However, in our simulations there was a difference in r_A_ following each selection cycle. In the Sim1000 HS families the observed r_A_ values were relatively lower compared to the Sim200 HS family simulations. While the r_A_ disparity between the populations is unclear, it should be noted that, Sim200 and Sim1000 were two independently simulated populations evaluated under different GE simulation systems, as described in “Material and Methods”. Populations evaluated under different GE systems, irrespective of the size of training population, produce different r_A_^63,64^. In forages, r_A_ is a result of genetic linkage and linkage disequilibrium (LD)^24,65,66^. As selection cycles progress the genetic linkage between the training and selection population declines, resulting in poor r_A_ as observed in our simulations (Fig. 5). To maintain higher r_A_ across multiple selection cycles, training populations need to be updated with new elite material or by including the families from previous selection cycles^58,61^. The implication of this strategy in HS breeding systems is yet to be investigated and would be considered in future studies.

Genetic variance within breeding populations provides the foundation for cultivar development^67^. In our simulations, there was a rapid decline in the percentage of genetic variance from selection C0 to C1, particularly for the A_*p*_W_*gs*_ and A_*gs*_W_*gs*_ methods and in the later selection cycles there was a steady decline in all three breeding strategies (Fig. 4). The rapid decline from C0 to C1 in the GS strategies is the result of utilizing both among (1/4 σ^2^_*A*_) and within (3/4 σ^2^_*A*_) HS family additive genetic variation, and this was reflected in the %∆G (Fig. 2). In addition, the polygenic architecture of the DM yield trait may be an underlying factor. Muleta, et al.^58^ reported similar trends, notably a rapid early decline in genetic variance, for a polygenic trait through their simulation of genomic assisted recurrent selection in sorghum (*Sorghum bicolor*). Cycles of continuous recurrent selection increase the frequency of favourable alleles and at the same time increase the probability of fixation of deleterious alleles. Selection pressure and population size have big impact on the allele fixation rates (Fig. 3). Our simulations demonstrated that smaller populations and high selection pressure will lead to higher allelic fixation rates earlier in the selection cycles. Allelic fixation rate is the direct measure of inbreeding in a population^27,68,69^. With decreasing population size and higher selection pressures the potential for inbreeding depression increases^55,68,69^. In cross pollinating species such as perennial ryegrass, which consists of heterozygous and heterogenous populations, inbreeding depression effects can be severe^70^. Our results re-emphasize the importance of larger training populations for GS implementation in order to maintain optimum levels of inbreeding rates and the genetic variance in a recurrent selection program.

In addition to having information such as heritability of key traits, the possibility of predicting the rate of decrease of their genetic variances and associated allele fixation rates, over multiple selection cycles, as shown in our study for HS families, also simulation studies by Esfandyari, et al.^61^ for FS families and Lin, et al.^55^ in a commercial breeding program, will enhance decisions on choice of breeding pool to achieve specific cultivar development goals, by deploying cost effective breeding strategies.

## Conclusion

Optimizing breeding program inputs for rate and cost-efficiency of genetic gain can be informed by simulation. Using mock data matrices constructed from empirically derived data, we demonstrated short- and long-term impacts of breeding strategy and integration of key technologies including genomic selection and phenomics on rate of predicted genetic gain for dry matter yield, a key economic trait, in perennial ryegrass. Our findings indicate these technologies offer substantial improvements in the rate of gain, and in some cases improved cost-efficiency per unit gain. The value of GS in exploiting within family additive genetic variation to increase genetic gain was demonstrated using both deterministic and stochastic simulation.

The application of GS in both among and within HS family selection in C2, provided a significant boost to total annual genetic gain across both cycles (C1 = 3 years and C2 = 1 year), even at low GS accuracy r_A_ of 0.12. Despite some reduction in genetic gain, using phenomics (LiDAR based mobile platform) to assess seasonal DM yield clearly demonstrated its impact by reducing cost per percentage gain relative to standard DM cuts.

The open-source software tools, DeltaGen and QU-GENE, offer ways to query and model the impact of breeding methodology and technology integration under a range of breeding scenarios and inputs in out crossing species including pasture species. This software expands the scope of tools available to breeders in decision support for breeding program design. The analyses reported in this paper can also be extended to major crop species using the genetic modelling capability for self-pollinating species, developed in both DeltaGen and QU-GENE.

## Supplementary Information

Supplementary Information.

## Data Availability

The datasets generated in this study are included as supplementary information files.

## Code availability

Software links to perform the deterministic and stochastic modelling were provided within this article.
