# Supplementary material for: Deterministic and stochastic modelling of impacts from genomic selection and phenomics on genetic gain for perennial ryegrass dry matter yield
Source: Sci Rep. 2021 Jun 24;11:13265. doi: 10.1038/s41598-021-92537-w (PMC8225875; doi:10.1038/s41598-021-92537-w)
Supplement: Supplementary file 1 — Supplementary Information. [file 41598_2021_92537_MOESM1_ESM.docx]

**File S1**

**Construction of mock data matrix used for modelling**

Construction of the mock data matrix for simulation consisted of the following steps:

1. Two existing growth score and herbage DM datasets were accessed. The first was 500 genomic selection training HS families reported by Faville et al., (2018). The second was another 500 HS families from trials reported by Arojju et al., (2020b) that had been evaluated across multiple contrasting environments (on the North and South islands of New Zealand, including sheep and dairy grazing environments), over two years, with data from three seasons per year,
2. Data from the entries were combined, to give 1000 HS families (3 replicates) across 2 locations, 2 years and 3 seasons (spring, summer and autumn) per year, resulting in a matrix of 36,000 data rows,
3. Growth score data, on a 0 to 9 scale, were used as a starting point to develop the matrix. In order to introduce a distribution within each score category, a random number between 0.01 and 0.90 was added to each data point within each replicate,
4. Using data from the HS family industry trials reported by Arojju et al., (2020b), seasonal calibration equations, based on herbage DM and growth score measurements, were developed for each season within each year (years 2 and 3), and each location,
5. the calibration equations (from d) were used to convert all the seasonal growth scores to DM yield (g m^-2^), which were converted to kg ha^-1^,
6. The final resulting data distributions are presented in supplementary figures S1a and S1b.


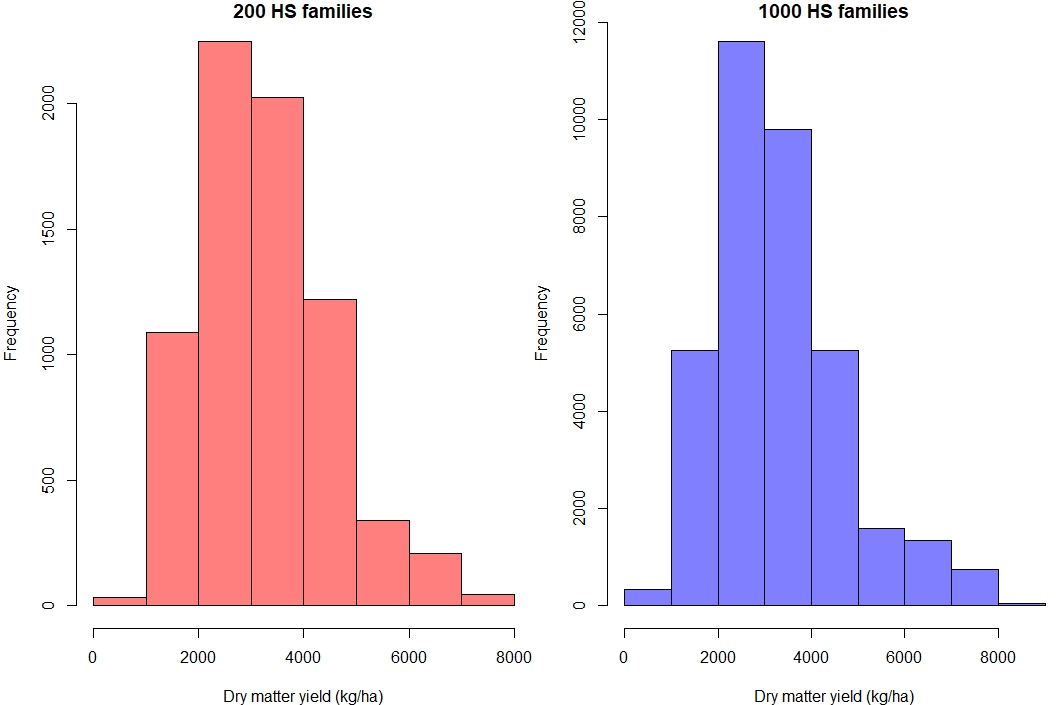


**Figure S1.** Distribution plots for the mock data sets of 200 and 1000 HS families of perennial ryegrass based on raw DM yield data. The 200 HS mock data had 4 zero values and no missing values. The 1000 HS mock data included 51 zero values and 45 missing values.

**Table S1.** Breakdown of costs* for the generation of genomic estimated breeding values (GEBV’s) per perennial ryegrass sample.

| **Step** | **Details** | **Cost per**  **sample ($ NZ)** | **Reference** |
| --- | --- | --- | --- |
| DNA isolation | Samples extracted at 96-well plate scale; four plates at a time. | 7 | Anderson et al., 2018 |
| GBS library creation | 384-plex GBS | 9 |  |
| GBS library sequencing | Illumina HiSeq 2500 using v4 chemistry^1^; one lane per 384-plex library. | 10 |  |
| Bioinformatics | Genotype calling using analysis pipeline based on TASSEL 5 software. | 10 | Faville et al., 2018; Glaubitz et al., 2014 |
| GEBV determination | Genomic relationship matrix using KGD software, GEBV's predicted using GBLUP. | 5 | Dodds et al. 2015 |
| Total |  | 41 |  |

^*^Conducted at AgResearch, Animal Genomics facility, Invermay Agricultural Centre, Mosgiel, New Zealand

**Table S2.** Cost structure of HS family field trial operation, herbage DM yield sampling and LiDAR phenotyping data acquisition (*Note: these values are estimates based on feedback from forage seed companies in New Zealand*).

| **Item** | **200 HS ($NZ)** | **1000 HS ($NZ)** |
| --- | --- | --- |
| Per DM sample | 7.50 | 7.50 |
| Per replicate | 250.00 | 500.00 |
| Location per year | 20,000.00 | 25,000.00 |
| Total general expenses across years, includes crossing of selected parents | 10,000.00 | 10,000.00 |
|  | **Calculating sample costs** | |
|  | **Manual** | **Phenomics (*Ph*)** |
| Time for cutting, drying, weighing herbage samples; 100 plots | 6 hrs | - |
| Time to scan 120 plots using *Ph* | - | 50 min |
| Cost ($) per FTE hour | 125.00 | 125.00 |
| Total $ per sampling 100 plots | 750.00 (6 hrs × 125.00) | - |
| Cost ($) per sample | 7.50 | - |
| Total $ per scanning 120 plots | - | 104.17 (0.833 hrs × 125.00) |
| Cost ($) per sample | - | 0.87 |


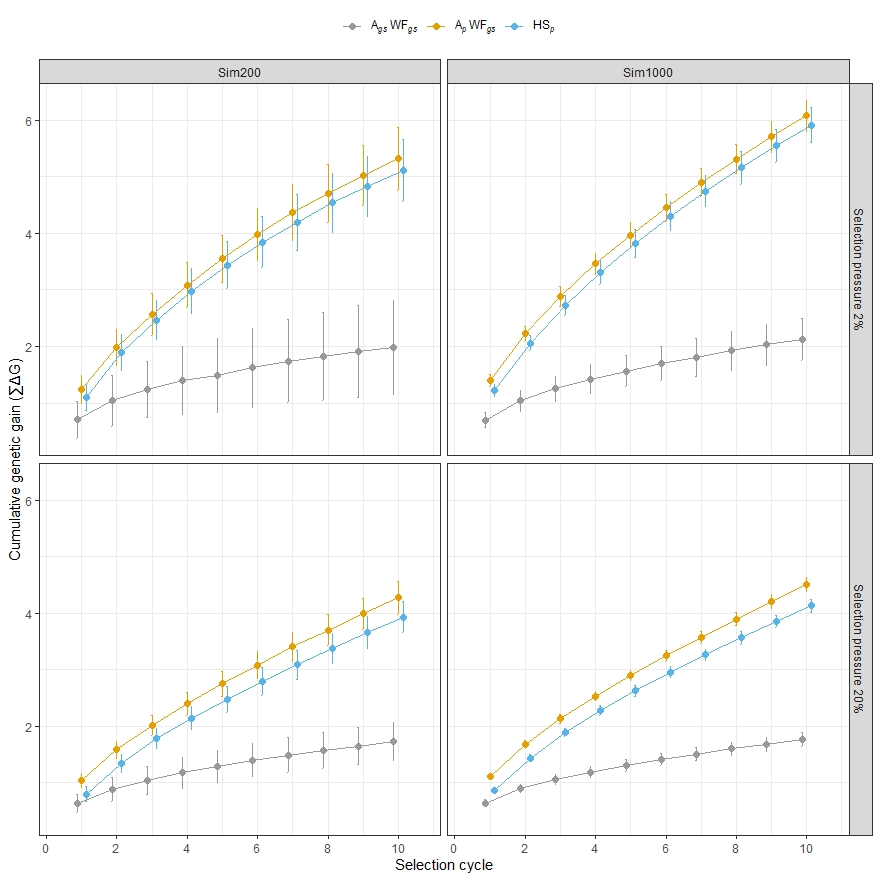

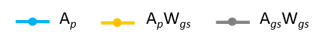


**Figure S2:** The cumulative genetic gain (∑ΔG) in Sim200 and Sim1000 training populations estimated across 10 selection cycles using three breeding strategies (A*_p_*, A*_p_*WF*_gs_* and A*_gs_*W*_gs_*). A selection pressure of 20% and 2% was imposed to select the best HS families. Within each family, for the A*_p_*W*_gs_* and A*_gs_*W*_gs_* strategies the top 5 or 50 individuals were selected and for the HS_P_ strategy 5 or 50 individuals were randomly selected to restore the initial number of parents for the next selection cycle.
